# Supplementary material for: Driver pattern identification over the gene co-expression of drug response in ovarian cancer by integrating high throughput genomics data
Source: Sci Rep. 2017 Nov 23;7:16188. doi: 10.1038/s41598-017-16286-5 (PMC5700962; doi:10.1038/s41598-017-16286-5)
Supplement: Supplementary file 1 — Supplementary information [file 41598_2017_16286_MOESM1_ESM.pdf]

Driver pattern identification over the gene co-expression of drug response in ovarian cancer by integrating high throughput genomics data
(Supplementary Material)

Xinguo Lu[1], Jibo Lu[1], Bo Liao[1], Xing Li[1], Xin Qian[1], Keqin Li[1,2]

1) College of Computer Science and Electronic Engineering, Hunan University, Lushan Nan Rd., Changsha 410082, China, 2) Department of Computer Science, State University of New York, New Paltz, NY 12561, USA.

S1 Table. initial modules list.

Modules	Genes
Module1	FBXO11;SLC26A6;KEL;ZBTB2;TFF1;CCBL1;SHD;F2;SCML2;THOP1;ADAR;PDE2A;NOX5;ITGB7;MYL4;RBL2;ENO3;GATA1;HBE1;PGC;ADAM23;RAB5A;APOB;NFE2L2;VGF;GALK1;TF;SPANXC;RHBG;RNF6;PGAP1;RHAG;PRH2;SERPIND1;DCT;ZNF620;DNTT;APOM;ASGR2;ZP1;KLF1;HBG1;APOA2;NCOA2;YES1;GYPC;NME4;DOT1L;CLTCL1;SERPINC1;AHSG;AMBP;SIX6;PRODH2;FGG;CGA;APOC3;HEMGN;NPTX1;SLC7A3;WNK4;KIAA1279;CES1;MLANA;RPS4Y2;C6orf141;TM4SF5;DUSP13;RBP4;PLAC4;ALAS2;ALB;JRKL;GATA3;CPNE7;ABCC2;MAGEA12;TFDP3;SOAT2;ALDOC;GDF3;WFDC2;CA5A;SERPINA7;MAGEA3;GDAP1L1;GPX2;POU5F1;ARID3A;HSPH1;DSCAM;RHOXF2B;ITIH3;PCSK9;RPS4Y1;CTSG;CPS1;BCAS1;AFP;AZGP1;EEF1A2;SPANXD;C4BPB;SPTA1;TYR;CPLX2;TTR;COX7A1;ISLR;ITIH2;APOH;IGFBP1;DPPA4;HBG2;C1orf64;ORM1;ORM2;TXLNB;FABP7;FABP1;PRSS3;L1TD1;FGL1;AGT;HSD17B2;CSAG1;PRSS2;MYL7;UTS2;CXorf48
Module2	EFCAB4B;CNNM3;OTX2;PNPLA7;STAT5B;DAZ2;SHBG;APBB1;MAP1A;PTK2B;ADAMTSL5;ZBTB10;LZTR1;XYLB;MADD;DAZ1;PCBP3;COPS7A;VWF;DDX6;MED17;CORO7;SV2A;DOHH;MIXL1;EIF2AK3;FBXL18;HEPACAM;ALG12;G6PD;CCL1;S100Z;ADAD2;KCNN1;PRPF19;RND2;NTRK1;PIAS4;LIM2;MPP2;NEK4;CREB3L3;DUSP9;HS3ST4;ZNF19;BRSK2;DFNB31;FXYD2;SPN;OSGIN1;PSG8;BAAT;NRIP3;LSM11;PSG4;CXorf23;F11;GNB3;IRX1;RASL10B;CNTNAP1;RIOK3;PKMYT1;CSNK1A1L;USP38;MASP2;TTTY13;AADACL4;FGFR4;TTN;C5orf38;LRRC4B;CHST13;HAR1A;FAM123A;ACSM2A;CCL16;SLC22A2;FGA;HILS1;SYDE1;CLDN15;WSCD1;STRN3;GNG13;SERPINF2;GPR63;CYGB;EXOC6;ANGPTL3;RNF38;C1orf187;TPTE;CSF3;THEG;LOC283693;RSPRY1;SLC8A3;EIF1AY;SLC9A5;AIFM3;CYP1A1;PKN3;ANKFN1;FSTL4;TPD52;CCR10;SPNS3;SYT3;KCNK6;ROBO4;SYN2;MEIS1;TAPT1;MDGA2;TNFSF9;PKLR;CRH;KIFC3;B4GALNT1;DDX3Y;OR5D13;GFI1B;FAM83G;PRDM9;TCEA1;RELL2;GCKR;RAB26;SLC39A5;ART4;NMNAT3;CDH9;GLYCTK;C2orf48;SMAD7;CETP;AMTN;SYNGR3;HRC;SOCS6;PAPPA;SFTPA1;CPN1;MAPK12;CORO6;HGFAC;PAX3;PBX1;SHC4;NLGN4Y;GGT6;KCNK12;LTB4R;ZDHHC22;SOX10;DLX1;CNTNAP4;TSGA10IP;MYPN;SCGB3A2;NR2E1;UPK1A;IL23R;RHD;CDH7;GLRA3;RALB;FTCD;ZNF488;APOA5;SLC5A11;NGFR;HSF4;AURKC;SLC2A2;RAET1E;RHEBL1;CCDC141;OR51I1;C1orf61;SLC18A1;HYAL1;NDST4;OR1D2;PRTG;MYH2;C8B;CD34;ANKS4B;PROC;PRAMEF8;IGFBPL1;SMAD4;MRGPRX3;SULT2A1;FUT5;USH1C;SMOC1;CALCR;MYOM3;PIWIL2;SYT6;SOX21;ZIM2;CYP2F1;MMRN2;KIAA1804;CACNA1H;GATA2;CDA;SERPINA4;PRODH;NOTUM;HBQ1;KLRC4;POU4F1;FSD1;TSPAN18;C3orf58;NKD1;TMEM171;PAH;PLP1;SLC30A10;MAGEB2;RLBP1;SLC17A2;PLA2G12B;XKR3;MAP2K7;SMCR5;SERPINA10;TRPV6;KRT34;F5;NOG;CLEC14A;TERT;FETUB;RAB3B;NR0B2;HAND1;IRF6;SLC38A5;CPA4;SERPINA11;SLC45A2;FAM9B;SLC30A3;GYPE;C8A;XPR1;TMEM145;SLC12A3;PTPRZ1;LCTL;BMP5;SERAC1;PRDM7;CLCA2;RNF138;WNT10B;CREG2;C8orf22;PTGFR;SULT2B1;DCX;MAPK4;KLB;HPD;MS4A3;NLRP11;CYP19A1;GALR1;ESX1;ADCY4;MYO1A;OR51B5;CDH1;SERPINA6;PRAP1;ISX;PAQR9;TRPM1;LBX2;DRD5;IGF2BP1;GCNT1;CHRNA9;PLAC2;CEACAM5;SPRR3;ADH4;SCGN;TLR5;KRTAP3-1;ARG1;KCNJ2;ACSM2B;KRTAP1-5;MIA2;XBP1;NEFM;SGPP2;DLX2;FGB;CDH4;C1orf210;SPINK4;MSMB;ABCC4;EMR1;BTLA;TFAP2B;VIL1;CPEB1;GABRB1;DQX1;NR1H4;ATP8B3;CLDN14;ALDH1A2;KCNE3;TRIM49;EFNB2;HOXC13;BLK;FGF19;NEFL;LOC

	441601;NDST3;GJA4;ODAM;TSPY2;PNLIPRP2;BTBD16;POU3F2;ONECUT1;TAC3;CHIT1;TMED6;GYPB;TYRP1;EGFL6;NPPB;DNER;TUBAL3;IRX2;LYVE1;SLC6A4;FOXG1;VIT;PRDM13;LRRTM1;LGR6;C16orf73
Module3	PRKG1;OXT;MEIS3;VAT1;ILK;SLC2A14;HLX;ADAMTS16;KERA;SH3BGRL3;SMPD1;MCHR1;BCAR1;NID1;VGLL3;EMP3;ELK3;KLF2;ACTC1;ALDH1B1;ID3;SLC12A4;LRRN3;EMILIN1;TFPI;ITGA5;OR7E24;ADAMTSL1;ETS1;ASGR1;CLEC3B;FHL3;GALNTL2;FAM126A;HAS1;HTR2A;XYLT1;TCP11L1;TLL1;CACNA1C;FEZ1;EHD2;CMTM3;C7orf10;CREB3L4;TPM1;PDLIM5;GPR68;CRISPLD2;SREBF1;KATNAL1;FSTL3;LATS2;RNF175;DGKI;CHSY1;PRND;MSC;TAS1R1;ZCCHC5;NKAPL;TGFB1I1;CBLN4;LIX1L;SLC22A3;F2RL2;TM4SF19;ZMAT3;SH3PXD2A;GREM2;SPOCD1;GPR1;EPHA2;IGFBP6;ELL2;TNC;EFEMP2;MGC45800;DACT3;FGF7;ACTA1;A1BG;SLC2A3;EXTL1;AQP1;RANBP3L;AKT3;ANGPTL2;SVEP1;CPNE8;CCL26;DOCK11;PMP22;NUAK1;GOLIM4;ANKRD29;THBS1;GFRA1;CNN2;PHLDA1;PPAPDC3;LMOD1;ZEB1;PSD3;FBN1;TRPA1;PLXNC1;PTRF;SLC16A7;ACAN;IGFBP4;FGF1;HSPB7;SERPINB7;FGF2;AFAP1;RBMS3;ENPP1;GRID1;GADD45B;MFSD4;DFNA5;RCAN1;AOC3;BDKRB1;PLXDC1;STX2;FOXF1;PTGER3;PROCR;COL8A1;ANTXR1;AK5;GALNT5;ECM1;OLFML3;RGS17;GNG11;TIAM2;SNAI1;DNAJB5;TGM2;TGFBI;APCDD1;VEGFC;GFPT2;PCOLCE;ANGPTL7;SPHK1;PDGFRA;ETV1;C16orf45;ZNF365;NBL1;TPM2;MEOX2;ODZ3;ISL1;ZNF215;PRKD1;COPZ2;PDZRN4;BACH2;RNF128;GAP43;FSTL1;NCAM2;THY1;HAND2;ADAMTSL3;LRRC15;MAF;F2R;BMP4;PHLDB2;GHR;DEPDC7;CDH13;BASP1;PLAC9;FLNC;SPOCK1;BNC2;BAI3;BMP2;SEMA3C;SEMA3D;AEBP1;KCNE4;NT5E;TNFSF4;JAM2;TAGLN;ACTA2;NEXN;LSAMP;RGS16;PRRX1;TWIST1;VDR;ZFHX4;HAS2;ST8SIA1;PDLIM3;CLSTN2;MXRA5;TMEM45A;CCDC80;SERPINE1;CNTN4;ADAM12;CDR1;GREM1;COL5A1;COL24A1;TIMP3;OMD;TPBG;EFEMP1;GDF6;GEM;NUDT11;SERPINF1;PLN;SALL4;CHRNA3;RGS2;OSR2;TBX18;DPT;FN1;IGF1;HS3ST2;EBF3;NUDT10;MAGEL2;ACTG2;ITGA11;ANGPTL1;SORCS2;STMN2;C1QTNF3;PDPN;PLAU;MFAP2;PPEF1;CH25H;MFAP4;MOXD1;CNN1;GAS1;SCG2;DSC3;COL5A2;BCHE;DKK1;CALB2;CILP;VCAN;CDH11;NDN;PDE1A;PPAPDC1A;DCN;ALPK2;SFRP1;CTHRC1;CCL11;SCRG1;COMP;LUM;HSPB3;ZNF521;INHBA;PTGIS;NNMT;THBS2;ITGBL1;GJB2;OGN;ASPN;SFRP4;IGFL2;LIX1;FAP;SFRP2;COL11A1;POSTN
Module4	ATPAF2;FOXD4L1;GNG8;BTN2A1;CNIH2;PRKDC;OAZ2;MCAT;RFNG;HMG20B;GOLGA4;GOLGB1;PRKAR1B;TAOK2;PIP5K1C;ZRANB1;ERGIC2;FAM96B;CLDN17;DDX28;BSG;POLRMT;ARL2;NAPB;FAM43B;C19orf24;NDOR1;LDB1;TXNL4A;SPIRE2;WDR18;KLF14;FAM108A1;ATP9B;CEP192;ZNF131;NCL;ZNF391;YIPF3;PROKR1;HBM;UXS1;MAP3K4;KCNAB3;DIRAS1;C10orf137;MKNK2;BCL7C;MEX3C;HNF1A;NDUFS7;FGF22;PFKL;LMAN1;KLF16;IL17RC;SYNGR4;NUP153;SS18;FOXC2;PASK;CFDP1;ALDH5A1;FAM70B;TRIP12;ZNF182;SACS;PIGW;UTF1;ZNF135;ABCA7;ZNF784;IRX4;SF3A2;AMMECR1;PHKA1;C21orf56;SOX17;SOX8;PGF;GRIN2C;SPPL2B;ZNF75A;CHORDC1;P2RY4;SNX8;HCN2;SLC34A3;ISOC1;PFKFB4;TLX2;IAPP;ATAD3C;GNG7;FUS;TCEAL3;HNRNPA2B1;TCEAL6;MGC45922;FOXB1;UCN;LIN7B;SLC4A8;HIRA;SLC22A18;ADRBK2;RNF145;TEPP;FBXL8;ITGB1BP3;CREBZF;CADM3;KCNMB3;FAIM3;AFG3L2;GPR151;KISS1R;SLC39A6;PGRMC1;MMP17;TM9SF2;ELMO3;GPR78;DOCK9;HAGHL;GPR20;PARD6A;MAFA;TCEAL5;ZSCAN10;CACNA1E;GAMT;GALNT9;LTC4S;AMH;HES7;C16orf74;FOXD1;HRK;CRYAB;HIST1H4L;NRN1;CMBL;SCGB3A1;ADAMTS1
Module5	FBXO11;SLC26A6;KEL;ZBTB2;TFF1;CCBL1;SHD;F2;SCML2;THOP1;ADAR;PDE2A;NOX

	5;ITGB7;MYL4;RBL2;ENO3;GATA1;HBE1;PGC;ADAM23;RAB5A;APOB;NFE2L2;VGF;GALK1;TF;SPANXC;RHBG;RNF6;PGAP1;RHAG;PRH2;SERPIND1;DCT;ZNF620;DNTT;APOM;ASGR2;ZP1;KLF1;HBG1;APOA2;NCOA2;YES1;GYPC;NME4;DOT1L;CLTCL1;SERPINC1;AHSG;AMBP;SIX6;PRODH2;FGG;CGA;APOC3;HEMGN;NPTX1;SLC7A3;WNK4;KIAA1279;CES1;MLANA;RPS4Y2;C6orf141;TM4SF5;DUSP13;RBP4;PLAC4;ALAS2;ALB;JRKL;GATA3;CPNE7;ABCC2;MAGEA12;TFDP3;SOAT2;ALDOC;GDF3;WFDC2;CA5A;SERPINA7;MAGEA3;GDAP1L1;GPX2;POU5F1;ARID3A;HSPH1;DSCAM;RHOXF2B;ITIH3;PCSK9;RPS4Y1;CTSG;CPS1;BCAS1;AFP;AZGP1;EEF1A2;SPANXD;C4BPB;SPTA1;TYR;CPLX2;TTR;COX7A1;ISLR;ITIH2;APOH;IGFBP1;DPPA4;HBG2;C1orf64;ORM1;ORM2;TXLNB;FABP7;FABP1;PRSS3;L1TD1;FGL1;AGT;HSD17B2;CSAG1;PRSS2;MYL7;UTS2;CXorf48
Module6	NLRC5;CD226;PIK3CD;PPM1M;FCRL3;C7orf41;SH2B3;P2RX7;COTL1;RPS27L;CACNA2D4;CATSPER1;LAT2;CCL23;TNFRSF10C;BMP2K;HERPUD1;MYO5A;GPR114;EBI3;PFKM;DPEP2;LILRA2;KIAA0513;HGF;TNFSF12;IRF8;APOL4;CORO1A;CLEC7A;CD274;LGALS9;GAB3;HAMP;BTN3A3;LGALS12;TDRD7;SEMA6C;LILRB1;RAC2;APOC2;RAB32;PSMB10;CYB5R4;CSF1R;CCL3;LAX1;CHRNA6;GNG12;DENND1C;IL32;PAG1;PLEK2;CECR6;SPECC1;GNB4;MEF2C;ARRDC2;LY86;PTPRO;CD302;BTK;STAT1;RNASE2;IL21R;CD86;SNX10;CD33;ARNT2;HMHA1;OLR1;PNMT;VAV1;CCL4;CCL17;RNASE6;CCDC109B;VMO1;KCTD12;IGSF21;APBB1IP;CFD;FXYD1;SAMSN1;EVI2B;FCGBP;CX3CR1;LY96;ANPEP;TPK1;APOC1;DAPP1;HLA-DQA1;CCL2;HLA-DQA2;IL18;FBXO32;BATF2;TNFSF10;CCL5;DDX25;EVI2A;DHRS9;CLEC2B;CCL8;IL15;GPX3;IL7;CCL18;GBP3;C10orf82;CD38;C3;CCL7;CCL13
Module7	ZNHIT3;ANKRD23;CENPT;ACTL6A;FXR1;DNAJC19;C6orf120;UNKL;MRPL47;OPA1;UBE2F;MUSTN1;PIK3CA;MFN1;SPCS2;MAD2L1BP;ZNF639;GMPS;C5orf22;LCAT;KPNA4;YIPF6;SENP2;RAB2A;RAB1A;ACSL3;DNAJB11;SLC33A1;PARL;WDFY1;ACVR1;TBL1XR1;GFM1;FNDC3B;SELS;NDUFA10;IER3IP1;SMC4;MYNN;RQCD1;MAGEF1;IMPAD1;NDUFB5;SERP1;TM2D3;PHACTR4;SSR1;PDCD10;PANK3;C3orf33;ZFHX3;ARPM1;UBE2E1;TMEM30A;AP1S3;ECT2;B3GALNT1;EID3;MAGEH1;SERPINI1;FRK;GPR160
Module8	C1orf201;CCDC104;CCDC113;LRRC27;C11orf74;HYDIN;TNFAIP8L1;C16orf48;ARL6;TEKT4;RFX2;WDR54;NEK10;GAS2L2;TEKT3;C1orf173;C22orf15;EFCAB2;CPXCR1;DUOX1;MNS1;DYX1C1;KCNE1;CACNG6;LRRC34;DNAH2;C8orf34;IQUB;CCDC17;LRRC46;RSPH10B;TSNAXIP1;TRIM55;LRRC48;CCDC89;CCDC135;DNAH10;ZNF214;STK33;WDR49;MEIG1;C10orf107;CCDC65;NME5;FAM92B;CXorf41;RGS22;TPPP3;SPATA4;C11orf70;C15orf26;ARMC4;C1orf114;AKAP14;WDR16;SRD5A2;C1orf87;DYNLRB2;TTC29;WDR69;ARMC3
Module9	PRKCG;RPL23;FAM138F;RPL19;COPB2;HSPA5;MRTO4;AARS;CCL24;HGSNAT;DYNC1LI2;PFDN5;NBPF15;TUG1;F11R;MRPL45;DAZAP2;EHMT1;ZNF593;AP3D1;NLE1;GLG1;RPL12;HHLA1;RPS15;OMP;UBC;LCE3A;HSP90B1;TAS2R9;MED1;RPL23A;RPL6;LASP1;RPL32;MRPL1;NTHL1;RPL36A;ATP5D;MRPL54;RPL27;HABP4;RPS21;RPS15A;RPL38;RPS7;SEMA4D;GPHB5;LIAS;AFMID;DDR1;OR10A7;RPL31;NPM3;RPL36;RPL34;CHD3;PDHA1;RPL29;RPL24;STARD9;ANKRD2;TOMM7;CDKL3;RPL35A;BEST4;HSD11B1L;SPAM1;TLE1;HMGCS1;RPS18;RPL39;RPS3A;SLC27A5;SOX4;SHROOM2;TAF15;ZNF556
Module10	PSMD6;KIAA2026;CBWD5;EXOSC3;CBWD6;ARPC5L;ATP6V1G1;UBAP2;CDC37L1;SM

	C5;GLRX5;FAM122A;C9orf64;ZDHHC21;FUNDC1;GINS3;KIAA0020;RBBP7;CBWD1;PPAPDC2;DNAJA1;USP1;PJA1;RMI1;C9orf82;ERMP1;MTM1;NFIB;VLDLR
Module11	PER1;PPP1R15A;RHOB;KLF4;KLF6;NR4A1;EREG;JUNB;HBA2;NR4A3;CTGF;ATF3;HBD;DUSP1;ZFP36;CYR61;FOSB;FOS;EGR3;EGR1;IL6;EGR2;AREG
Module12	HSP90AB1;CTNNBL1;PSPC1;SMARCAD1;TRADD;RSAD1;CKLF;CPNE1;WDR35;MT1X;PALM;MT2A;ASPHD1;MT1B;MT1G;MT1JP;LGALS2;MT1H;MT1M;MT1E;TFPI2
Module13	TAF12;POLR2E;AKR7A2;LDLRAP1;CDC34;AKR7A3;RGS19;RPS6KA1;AP1S1;CASP9;ARFRP1;TNIP2;HMGCL;ALDH4A1;WASF2;LYPLA2;TRAF7;RPL11;NOC2L;PABPC4;C1orf38
Module14	PRKRA;GGPS1;RBM34;DUSP12;C1orf131;FH;EGLN1;TOMM20;EXOC8;CD46;C1orf31;ZBTB7A;ARID4B;SCCPDH;CPOX;ADSS;TAF5L;ACBD3;TTC13;SRP9
Module15	LYL1;MYO1G;ZAP70;GZMM;GNA15;SCO2;MATK;MFNG;KLRK1;CD8B;RAB33A;CD3D;CBFA2T3;BCL11B;KLRC2;KLRD1;KLRC3
Module16	IL1F10;WDR87;PIK3R5;OR4S1;MYH8;SLC10A1;PPM1F;SPZ1;ARHGAP22;VPS18;ZCCHC13;CABP7;PCSK4;TEX12;CGB1;C9orf47
Module17	CHAF1A;YIF1B;CCNF;WDR62;MXD3;BCL2L12;PPIH;LMNB2;GTSE1;AURKB;CENPO;CDC20;GINS4;MGC21881;C1orf135;ZYG11A
Module18	BTBD2;GNA11;MAP2K2;DCTN1;PNPLA6;GTF2F1;ZNF358;PLD2;DUS3L;TRIP10;CBX6;NFIC;EEF2;MAN2B1;TIMM44;SH2D3A
Module19	ZNF707;PHF20L1;ZNF34;FAM91A1;HSF1;PUF60;ZNF7;CHRAC1;ZNF16;EXOSC4;SCRIB;TIGD5;PTP4A3;SQLE;C8orf51
Module20	ARMC6;FBXO24;TMEM161A;PGPEP1;CCDC124;DDX49;MAST3;UBA52;FAM125A;STX10;C19orf60;C19orf44;FBXW9;MAST1;RPL18A
Module21	JTB;UBFD1;FDPS;PEX19;FDPSL2A;PIGM;KARS;ATP6V0D1;ADAT1;MLYCD;SYNPO2L;ZNF689;CCNDBP1;PANK1
Module22	RIN1;SERPINA1;ST3GAL1;CHI3L2;CYP24A1;VNN1;SAA4;HP;LRG1;PI3;SAA2;S100A9;SAA1;HPR
Module23	MAGEA5;SSX5;SSX3;DSCR4;SSX2;SSX1;SSX7;MAGEA1;MAGEA10;MAGEA8;MAGEA11;DSCR8;MAGEA9
Module24	CAMK1G;SLC4A4;WFDC1;PCSK6;GREB1;RBP7;LAMA2;GATM;CCDC48;DLK1;WNT2B;FOXL2;TSPAN8
Module25	EXOSC9;IDH2;SNRPA1;POLG;FANCI;CENPE;GINS2;RAD51;DIAPH3;MND1;NEIL3;GGH;NMU
Module26	CCL15;MRAP;SCARA5;PI16;ASPA;ADH1B;PPARG;CD36;SV2B;PLA2G2A;TIMP4;FABP4
Module27	KRT80;KRT5;ANXA8L2;KRT6C;TGM1;KRT6B;GPR87;KRT6A
Module28	HIST1H3A;SH3BP5L;H3F3A;RAB3GAP2;MIA3;B3GALNT2;PARP1;MCM3
Module29	POLR3A;AKT1S1;RPL18;RPS11;RPL13A;RPL26;C17orf48;RPS24
Module30	SAMD10;CRCT1;KRTAP5-8;MAB21L2;DIRC1;PRSS36;IL27
Module31	HOXB4;HOXB7;HOXB3;HOXB5;HOXB2;HOXB8;HOXB6
Module32	SERGEF;GNB2L1;EIF3F;IPO7;RPS13;RPLP2;RPL27A
Module33	UGT2B4;UGT2A3;UGT2B10;CD200;UGT2B11;UGT2B7
Module34	PAGE1;COX7B2;PAGE5;MAGEC1;PAGE2;MAGEC2
Module35	ANKRD27;C19orf40;POP4;C19orf12;CCNE1;CDKN2A

Module36	HOXA2;HOXA7;HOXA5;HOXA9;HOXA4
Module37	CD1B;CD1E;CD1C;S100B;CD1A
Module38	SNAPC2;TRAPPC5;NDUFA7;CD320;RPS28
Module39	SUPT16H;APEX1;METTL3;DAD1;PARP2
Module40	PCDHB15;PCDHB14;PCDHB8;PCDHB16;PCDHB5

S2 Table. List of initial modulators for the mutation genes.

GOLIM4;KCNMB2;MRPL47;CCDC39;SERPINI1;WDR49;LOC646168;EGFEM1P;TNFSF10;MECOM;ECT2;SPATA16;NLGN1;TBL1XR1;ZMAT3;PIK3CA;ZNF639;USP13;PEX5L;PDCD10;MIR551B;MYNN;LRRC34;LRRIQ4;TERC;LRRC31;ARPM1;LOC100128164;SAMD7;GHSR;NAALADL2-AS3;MIR4789;KCNMB3;MFN1;GNB4;ACTL6A;NDUFB5;TTC14;DNAJC19;FXR1;ASAP1;KCNQ3;PVT1;ADCY8;OC90;PHF20L1;TG;ST3GAL1;ZFAT;KHDRBS3;POU5F1B;LOC727677;MYC;MIR1204;MIR1205;MIR1207;MIR1206;MIR1208;LOC728724;GSDMC;EFR3A;HHLA1;LRRC6;HPYR1;TMEM71;NDRG1;ZFAT-AS1;MIR30B;MIR30D;LOC286094;FLJ43860;GPAA1;TCEA1;FBXO11;ARMC4;ZNF20;ZNF707;CDCA8;FARSA;ADCK5;ZNF527;TRPC4AP;JRKL;SDHA;ENDOD1;YTHDF3;LAPTM4A;FBXL6;WISP1;SLA;STK3;FAM91A1;EED;IFT52;ZNF345;ARFGAP1;TIGD5;HSF1;SAMD9L;FAM66E;DEFB103A;SPAG11B;DEFB103B;DEFB4B;SPAG11A;DEFB4A;LOC100132396;BANP;JPH3;ZCCHC14;KLHDC4;MYH10;STX8;SRCIN1;ACACA;LINC00324;NTN1;GAS7;USP43;MYH13;MYH4;HS3ST3A1;ADORA2B;KSR1;KRT18P55;RAD51L3-RFFL;CCL23;AP2B1;GAS2L2;SLFN11;MYO19;AATF;SYNRG;TADA2A;FBXL20;LOC100131347;CWC25;PLXDC1;FBXO47;PSMD3;WIPF2;ERBB2;IKZF3;ALOX15B;ALOX12B;GUCY2D;MIR4314;PER1;ALOXE3;TMEM107;AURKB;VAMP2;MIR3676;C17orf59;MIR4521;RANGRF;ARHGEF15;PFAS;ODF4;SLC25A35;CTC1;RPL26;KRBA2;NDEL1;LOC100128288;RNF222;CCDC42;MFSD6L;SPDYE4;PIK3R6;DHRS7C;RCVRN;GLP2R;MYH8;MYH1;CDRT15P2;MEIS3P1;TBC1D26;ZSWIM7;UBB;SMCR5;C17orf51;WSB1;MIR4522;TBC1D3P5;LGALS9;NOS2;PPY2;CCL7;CCL2;CCL8;CCL13;TMEM132E;C17orf102;ZNF830;CCT6B;RFFL;LIG3;SLC35G3;RAD51D;UNC45B;FNDC8;NLE1;SLFN5;SLFN13;SLFN12;SLFN12L;PEX12;SLFN14;SNORD7;RASL10B;C17orf50;TAF15;MMP28;C17orf66;CCL14;CCL15;RDM1;CCL14-CCL15;LYZL6;CCL3;CCL18;CCL3L1;CCL3L3;CCL4L2;CCL4L1;TBC1D3C;TBC1D3B;TBC1D3H;ZNHIT3;TBC1D3G;GGNBP2;PIGW;DHRS11;MRM1;LHX1;MIR2909;C17orf78;DUSP14;DDX52;TBC1D3F;LOC284100;TBC1D3;LOC440434;MRPL45;SOCS7;GPR179;ARHGAP23;C17orf96;PIP4K2B;PCGF2;PSMB3;CISD3;MIR4734;MIR4726;MLLT6;RPL23;SNORA21;LASP1;C17orf98;LOC100505576;MIR4727;FLJ43826;CACNB1;ARL5C;STAC2;RPL19;MED1;CDK12;STARD3;PPP1R1B;NEUROD2;PGAP3;PNMT;TCAP;MIEN1;MIR4728;GSDMB;LRRC3C;ZPBP2;GSDMA;ORMDL3;SNORD124;THRA;MED24;CSF3;NR1D1;MSL1;CASC3;RAPGEFL1;RARA;GJD3;CDC6;ANKRD24;GNA15;IZUMO4;ABCA7;RNF126;KISS1R;REXO1;CSNK1G2;DOT1L;TLE2;GNG7;CELF5;MFSD12;NFIC;ZBTB7A;MATK;STAP2;PLIN3;UBXN6;DPP9;THEG;PPAP2C;MIER2;TPGS1;ODF3L2;C2CD4C;MADCAM1;SHC2;GZMM;POLRMT;CDC34;BSG;HCN2;FGF22;PALM;C19orf21;PRSS57;FSTL3;PTBP1;AZU1;MED16;PRTN3;CFD;MIR3187;ELANE;LPPR3;MIR4745;R3HDM4;C19orf6;WDR18;CNN2;GRIN3B;ARID3A;HMHA1;SBNO2;POLR2E;GPX4;EFNA2;CIRBP;ATP5D;C19orf24;C19orf26;STK11;MIDN;CIRBP-AS1;MUM1;NDUFS7;DAZAP1;GAMT;RPS15;REEP6;PCSK4;MEX3D;C19orf25;PLK5;APC2;ADAMTSL5;TCF3;UQCR11;MBD3;ATP8B3;MIR1909;LOC100288123;ONECUT3;CSNK1G2-AS1;SCAMP4;ADAT3;MKNK2;MOB3A;BTBD2;AP3D1;C19orf35;JSRP1;PLEKHJ1;SPPL2B;MIR4321;OAZ1;LSM7;AMH;MIR1227;LINGO3;TIMM13;GADD45B;LMNB2;TMPRSS9;SLC39A3;DIRAS1;ZNF555;THOP1;SGTA;ZNF554;ZNF556;TLE6;ZNF57;ZNF77;GNA11;AES;S1PR4;NCLN;FZR1;DOHH;C19orf77;C19orf71;GIPC3;HMG20B;TBXA2R;C19orf29-AS1;TJP3;APBA3;MRPL54;RAX2;ZFR2;ATCAY;EEF2;PIAS4;SNORD37;MIR637;DAPK3;ITGB1BP3;CREB3L3;SIRT6;MAP2K2;EBI3;FSD1;SHD;T

MIGD2;CCDC94;SH3GL1;MPND;CHAF1A;MIR4746;PLIN4;LRG1;HDGFRP2;SEMA6B;PLIN5;C19orf10;TNFAIP8L1;LOC100131094;MIR7-3HG;TICAM1;FEM1A;MIR7-3;LOC100128946;XPNPEP3;FAM19A5;NFATC3;LOC283867;TMCO7;PLA2G15;CDH1;SMPD3;ESRP2;SLC7A6;PRMT7;SLC7A6OS;HAS3;GGA1;TMEM184B;PLA2G6;TRIOBP;EIF3L;CBY1;DMC1;CACNA1I;CYTH4;ELFN2;CARD10;LGALS2;CDC42EP1;SH3BP1;LGALS1;NOL12;PDXP;ANKRD54;GCAT;MIR659;MIR658;H1F0;POLR2F;MICALL1;C22orf23;MIR4534;PICK1;BAIAP2L2;SLC16A8;MAFF;LOC400927;DDX17;KCNJ4;KDELR3;LOC646851;DNAL4;SUN2;GTPBP1;TOMM22;JOSD1;NPTXR;CBX6;APOBEC3B;APOBEC3A;APOBEC3C;APOBEC3D;APOBEC3F;CBX7;APOBEC3G;MGAT3;ATF4;SMCR7L;RPS19BP1;MYH2;SOX10;SLC12A3;FAM120B;PPP2R2A;LONP1;CAMK2D;CSNK1E;ASS1;GALR3;APOBEC3H;RB1;CCDC135;MFNG;C9orf82;SOSTDC1;RRM1;MT1M;SLC18A1;EIF4G3;XRCC4;KLF16;APOL6;PIP5K1C;WDR16;CCL1;VPS33B;LOXL2;FAM108C1;HPS4;C19orf29;DNAJC16;C1orf172;FGL1;TOP2A;GRB7;USP48;UBE4B;ZCCHC9;SCAMP1;ZDHHC21;RAB28;MSR1;CCL16;FAM108A1;CCL11;DOK2;CCL4;HES7;CCL5;DHRS4;MDN1;HIATL1;PIK3R5;HNF1B;NUP43;TCP1;C6orf120;ZNF500;SF3A2;PIWIL2;APOB;BRCA1;BRCA2;COL6A6;CSMD3;DNAH3;DNAH8;FLG2;HSPG2;HUWE1;KIAA1109;ODZ1;RYR2;SLCO1C1;STAB2;SYNE2;ZFHX4;NEB;RB1CC1;SCN8A;CREBBP;DST;FAT3;FLG;HMCN1;LRP1B;LRP2;MUC17;NF1;PTPRZ1;SORCS3;SPTB;TP53;TTN;UBR4;MT-ND4;USH2A;HYDIN;RYR1;MUC16;

S3 Table. Modulator gene list after module network learning.

Modulator gene list							
MT1M	GOLIM4	C1orf172	AATF	LMNB2	C19orf24	LRRC31	RPS15
LASP1	JPH3	ADAMTSL5	MYC	PALM	TNFSF10	HMG20B	SOSTDC1
AMH	SLC18A1	HES7	C6orf120	FAM108A1	TIGD5	TLE2	WSB1
HNF1B	UBE4B	MRPL47	POLRMT	FAM91A1	SOX10	FBXO11	KLF16
PHF20L1	SLFN13	MYH2	CDC34	MED24	TBL1XR1	MFNG	MYH8
ARPM1	CCL11	HHLA1	NDUFB5	FGL1	DOT1L	MRM1	MRPL54
FSTL3	PIK3R5	CNN2	C9orf82	SBNO2	CCL8	ZNF707	CCL7
PDCD10	CCL5	GNA11	CCL4	KISS1R	KCNMB3	POLR2E	MFN1
FSD1	ECT2	CCL18	GAS2L2	CCL16	FGF22	RPL19	WDR16
SLC12A3	CCL23	C19orf10	RCVRN	SF3A2	GADD45B	RASL10B	WDR18
CCDC135	EEF2	PLXDC1	ARHGAP23	GZMM	LRG1	PPP1R1B	TCAP
BRCA1	HYDIN	APOB	MUC16	ZFHX4			

S4 Fig. List of dosage-sensitive and dosage-resistant genes and the scatter diagrams of these genes. We applied a local polynomial regression fitting model with the R package loess to the scatter diagram for 93 regulatory genes with 324 samples. DS threshold is set to (-0.25,0.25) as the filtering criteria for dosage-sensitive genes(DSGs) and the others were selected as dosage-resistant genes(DRGs). Therefore, we obtained 44 DSGs and 49 DRGs (see Supplementary Fig. S4). The LRG1, HYDIN, POLRMT and TNFSF10 gene data scatter diagram for CNVs or somatic mutations vs. Gene expression with local polynomial regression fitting model are shown in Fig. 3 in our main manuscript, in which HYDIN is derived from somatic mutations. Each red point represents an independent sample, the black line is the linear regression result, and the blue curve is the local polynomial regression fitting result. In Fig. 3A and Fig. 3B the slope of the black lines are close to 0, so the dosage sensitivity score are nearly equal to 0. In Fig. 3C and Fig. 3D the

slope's absolute value of the black line are above 1, the blue curves are mostly overlapped with the black line, so the dosage sensitivity score are above 1. It can be concluded from the graph that the genes for Fig. 3A and Fig. 3B are dosage-resistant genes, whereas the genes for Fig. 3C and Fig. 3D are dosage-sensitive genes. Here, we list another 10 scatter diagrams(related gene list: ADAMTSL5, APOB, ARHGAP23, ARPM1, AZU1, BRCA1, C19orf10, C19orf24, C1orf172 and C6orf120) of these 93 genes.

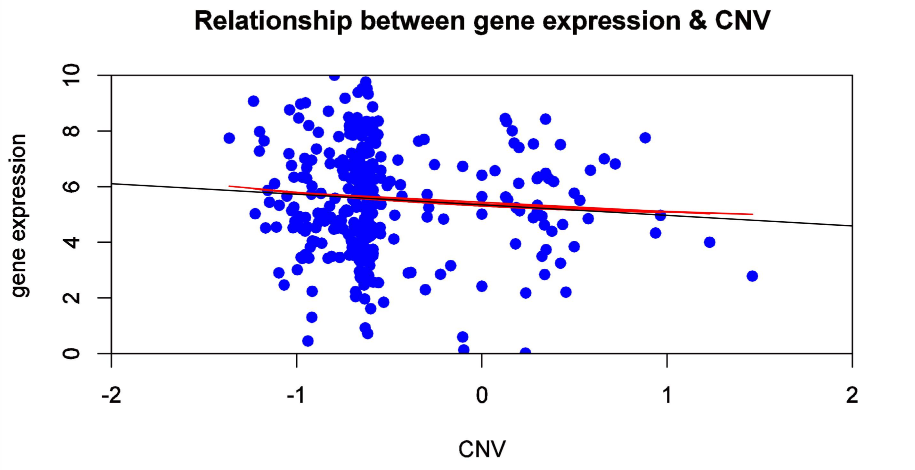

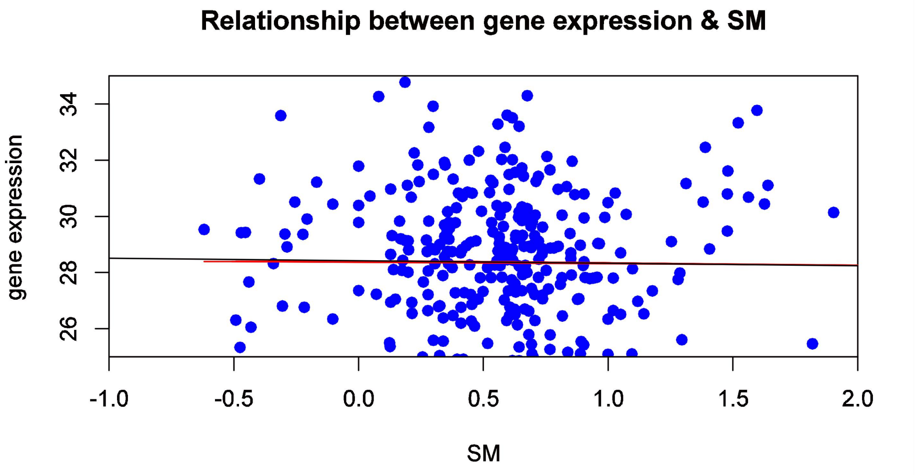

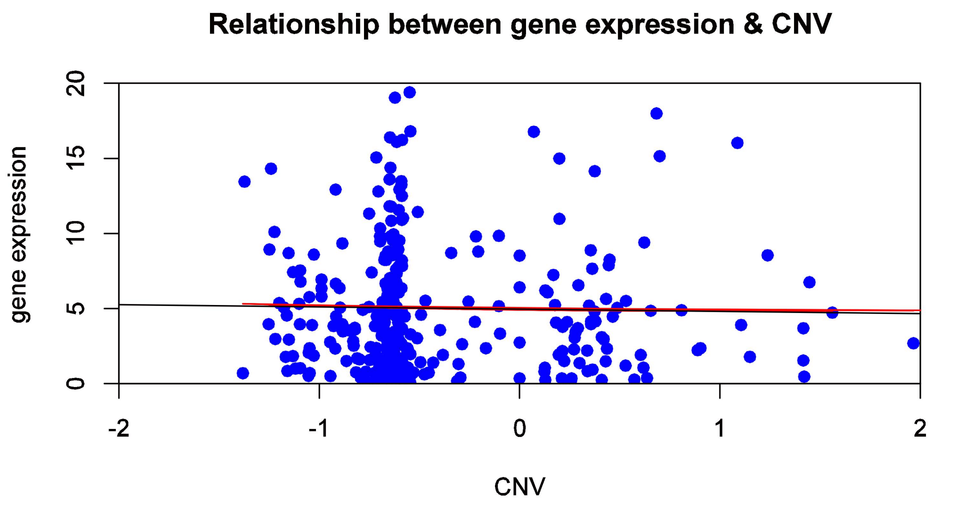
Relationship between gene expression & CNV
gene expression
0
5
10
15
20
-2
-1
0
1
2
CNV

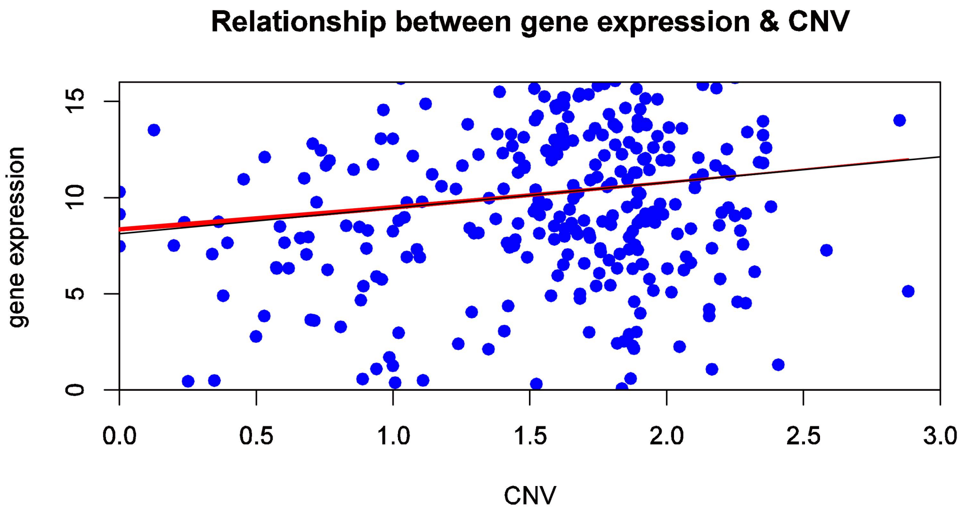
Relationship between gene expression & CNV
gene expression
0
5
10
15
0.0
0.5
1.0
1.5
2.0
2.5
3.0
CNV

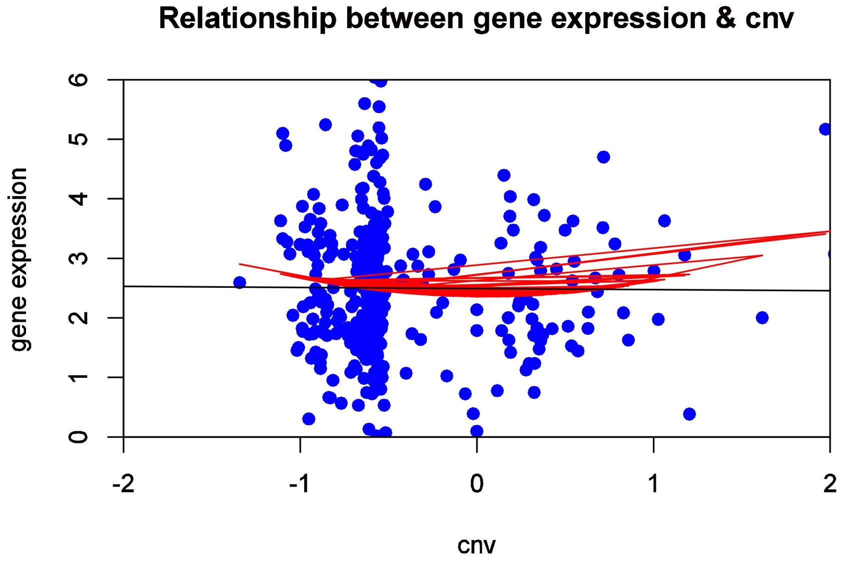
Relationship between gene expression & cnv
gene expression
0
1
2
3
4
5
6
-2
-1
0
1
2
cnv

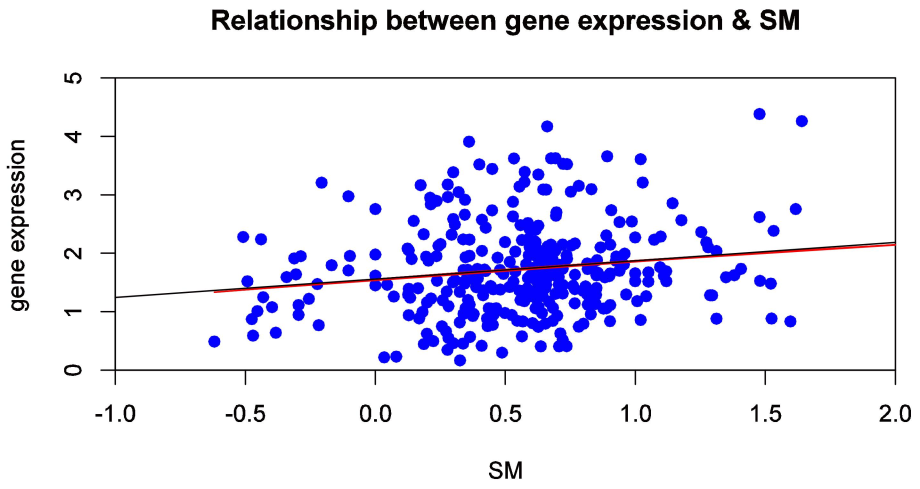
Relationship between gene expression & SM
gene expression
SM
0
1
2
3
4
5
-1.0
-0.5
0.0
0.5
1.0
1.5
2.0

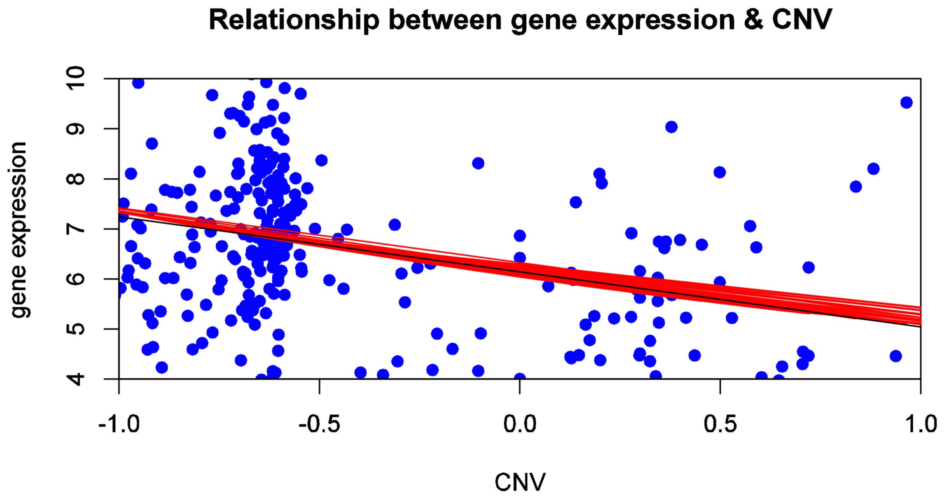
Relationship between gene expression & CNV
gene expression
CNV
4
5
6
7
8
9
10
-1.0
-0.5
0.0
0.5
1.0

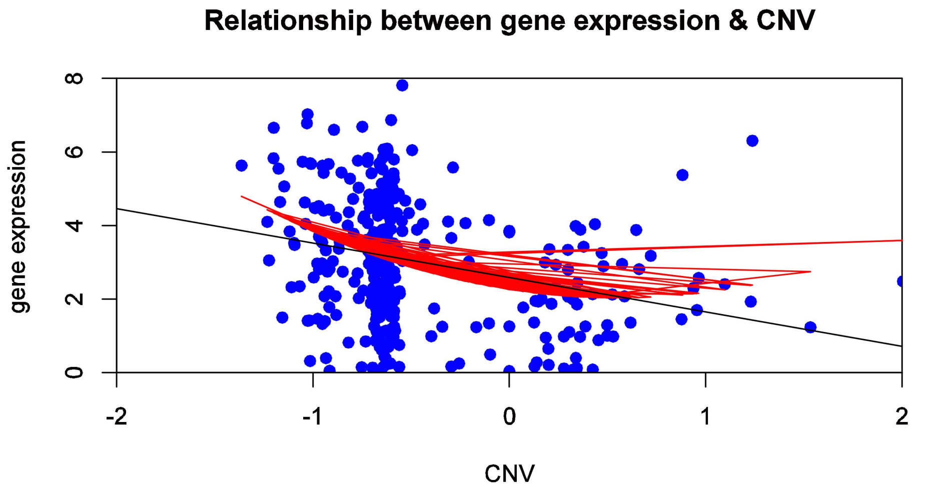
Relationship between gene expression & CNV
gene expression
CNV
0
2
4
6
8
-2
-1
0
1
2

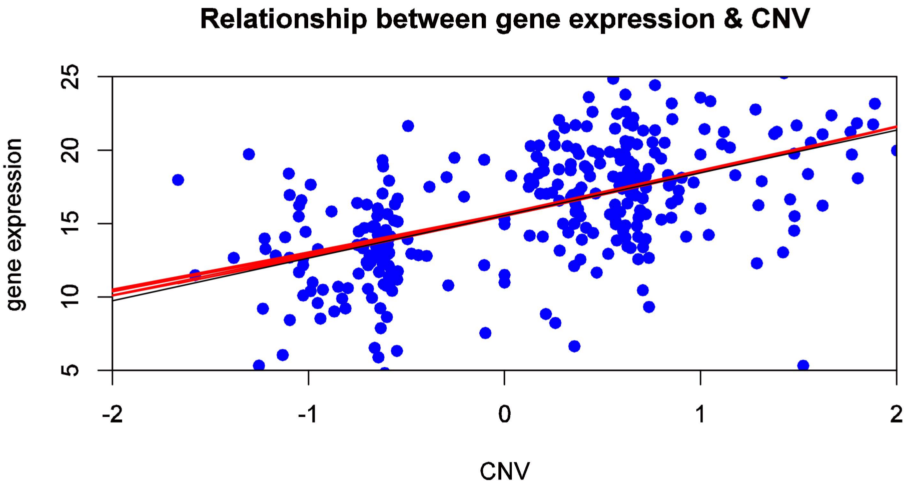

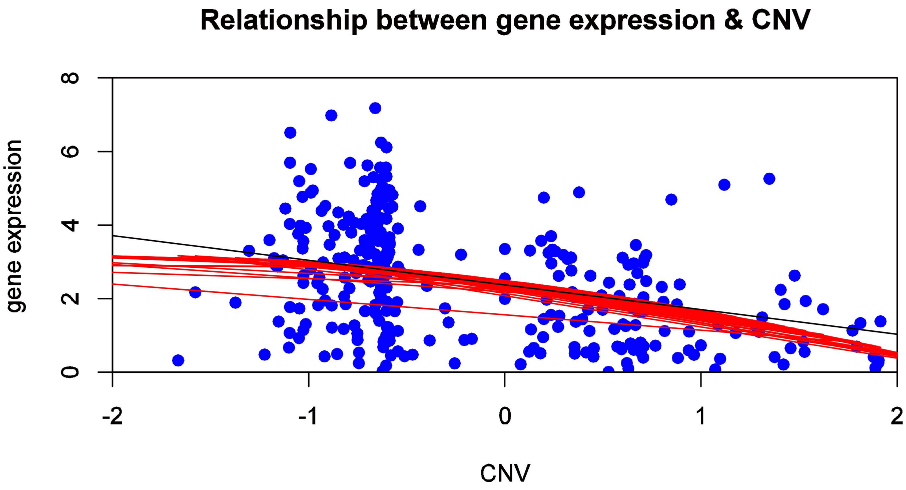

S5 Table. The GO term pathways enriched by DRGs.

Pathway ID	Description	Gene Ratio	BgRatio	pvalue	p.adjust	qvalue	geneID	Count
GO:0048247	lymphocyte chemotaxis	6/40	55/16672	3.43E-09	2.51E-06	2.12E-06	CCL11/CCL16/CCL18/CCL23/CCL8/CCL5	6
GO:0002548	monocyte chemotaxis	6/40	58/16672	4.76E-09	2.51E-06	2.12E-06	CCL11/CCL16/CCL18/CCL23/CCL8/CCL5	6
GO:007	mononu	6/40	73/166	1.95E-	6.86E-	5.80E-	CCL11/	6

1674	clear cell migration		72	08	06	06	CCL16/CCL18/CCL23/CCL8/CCL5	
GO:0070098	chemokine-mediated signaling pathway	6/40	80/16672	3.40E-08	6.95E-06	5.87E-06	CCL11/CCL16/CCL18/CCL23/CCL8/CCL5	6
GO:0072676	lymphocyte migration	6/40	81/16672	3.67E-08	6.95E-06	5.87E-06	CCL11/CCL16/CCL18/CCL23/CCL8/CCL5	6
GO:0030593	neutrophil chemotaxis	6/40	82/16672	3.95E-08	6.95E-06	5.87E-06	CCL11/CCL16/CCL18/CCL23/CCL8/CCL5	6
GO:0071347	cellular response to interleukin-1	6/40	85/16672	4.91E-08	7.40E-06	6.25E-06	CCL11/CCL16/CCL18/CCL23/CCL8/CCL5	6
GO:1990266	neutrophil migration	6/40	88/16672	6.05E-08	7.40E-06	6.25E-06	CCL11/CCL16/CCL18/CCL23/CCL8/CCL5	6
GO:0097529	myeloid leukocyte migration	7/40	151/16672	6.31E-08	7.40E-06	6.25E-06	CCL11/CCL16/CCL18/CCL23/CCL8/CCL5/AZU1	7

GO:0071621	granulocyte chemotaxis	6/40	97/16672	1.09E-07	1.11E-05	9.41E-06	CCL11/CCL16/CCL18/CCL23/CCL8/CCL5	6
GO:0071356	cellular response to tumor necrosis factor	8/40	250/16672	1.16E-07	1.11E-05	9.41E-06	APOB/CCL11/CCL16/CCL18/CCL23/CCL8/CCL5/BRCA1	8
GO:0097530	granulocyte migration	6/40	106/16672	1.84E-07	1.61E-05	1.36E-05	CCL11/CCL16/CCL18/CCL23/CCL8/CCL5	6
GO:0034612	response to tumor necrosis factor	8/40	268/16672	1.98E-07	1.61E-05	1.36E-05	APOB/CCL11/CCL16/CCL18/CCL23/CCL8/CCL5/BRCA1	8
GO:0070555	response to interleukin-1	6/40	110/16672	2.30E-07	1.65E-05	1.40E-05	CCL11/CCL16/CCL18/CCL23/CCL8/CCL5	6
GO:0030595	leukocyte chemotaxis	7/40	183/16672	2.35E-07	1.65E-05	1.40E-05	CCL11/CCL16/CCL18/CCL23/CCL8/CCL5/AZU1	7
GO:0050729	positive regula	6/40	115/16672	2.99E-07	1.98E-05	1.67E-05	CCL11/CCL16/CCL18/	6

	tion of inflammatory response						CCL23/CCL8/CCL5	
GO:0071346	cellular response to interferon-gamma	6/40	138/16672	8.78E-07	5.46E-05	4.61E-05	CCL11/CCL16/CCL18/CCL23/CCL8/CCL5	6
GO:0060326	cell chemotaxis	7/40	240/16672	1.46E-06	8.56E-05	7.23E-05	CCL11/CCL16/CCL18/CCL23/CCL8/CCL5/AZU1	7
GO:0050900	leukocyte migration	8/40	362/16672	1.92E-06	0.000106108	8.96E-05	APOB/CCL11/CCL16/CCL18/CCL23/CCL8/CCL5/AZU1	8
GO:0034341	response to interferon-gamma	6/40	159/16672	2.01E-06	0.000106108	8.96E-05	CCL11/CCL16/CCL18/CCL23/CCL8/CCL5	6
GO:0032103	positive regulation of response to external stimulus	7/40	261/16672	2.55E-06	0.000128219	0.000108255	CCL11/CCL16/CCL18/CCL23/CCL8/CCL5/AZU1	7
GO:007	positi	6/40	170/16	2.96E-	0.0001	0.0001	CCL11/	6

0374	ve regulation of ERK1 and ERK2 cascade		672	06	42233	20087	CCL16/CCL18/CCL23/CCL8/CCL5	
GO:0070372	regulation of ERK1 and ERK2 cascade	6/40	237/16672	1.98E-05	0.000910875	0.00076905	CCL11/CCL16/CCL18/CCL23/CCL8/CCL5	6
GO:0070371	ERK1 and ERK2 cascade	6/40	251/16672	2.74E-05	0.001206168	0.001018366	CCL11/CCL16/CCL18/CCL23/CCL8/CCL5	6
GO:0050727	regulation of inflammatory response	6/40	295/16672	6.75E-05	0.002850541	0.002406707	CCL11/CCL16/CCL18/CCL23/CCL8/CCL5	6
GO:0050918	positive chemotaxis	3/40	52/16672	0.000260624	0.010585327	0.008937173	CCL16/CCL5/AZU1	3
GO:0048245	eosinophil chemotaxis	2/40	11/16672	0.000304507	0.011909597	0.010055252	CCL11/CCL5	2
GO:0031349	positive regulation of defense response	6/40	396/16672	0.000334988	0.0126 3385	0.010666738	CCL11/CCL16/CCL18/CCL23/CCL8/CCL5	6
GO:007	eosino	2/40	15/166	0.0005	0.0210	0.0177	CCL11/	2

2677	phil migration		72	77811	40279	64271	CCL5	
GO:0009615	response to virus	5/40	304/16672	0.000760625	0.02677399	0.022605232	APOB/CCL11/CCL8/CCL5/AZU1	5
GO:0043410	positive regulation of MAPK cascade	6/40	471/16672	0.000836228	0.02848568 9	0.024050418	CCL11/CCL16/CCL18/CCL23/CCL8/CCL5	6
GO:0002544	chronic inflammatory response	2/40	22/16672	0.001257754	0.041505871	0.035043334	CCL11/CCL5	2
GO:0048246	macrophage chemotaxis	2/40	26/16672	0.001758868	0.056283782	0.047520299	CCL5/AZU1	2
GO:0006874	cellular calcium ion homeostasis	5/40	370/16672	0.001820676	0.05654805	0.047743419	CCL11/CCL23/CCL8/CCL5/JPH3	5
GO:0055074	calcium ion homeostasis	5/40	383/16672	0.002117117	0.06387644	0.053930766	CCL11/CCL23/CCL8/CCL5/JPH3	5
GO:0072503	cellular divalent inorganic cation homeos	5/40	395/16672	0.0024 2086	0.071011885	0.0599 5521	CCL11/CCL23/CCL8/CCL5/JPH3	5

	tasis							
GO:0045766	positive regulation of angiogenesis	3/40	120/16672	0.002958989	0.083262848	0.070298676	CCL11/LRG1/BRCA1	3
GO:0072507	divalent inorganic cation homeostasis	5/40	415/16672	0.002996201	0.083262848	0.070298676	CCL11/CCL23/CCL8/CCL5/JPH3	5

S6 Table. The GO term pathways enriched by DSGs.

Pathway ID	Description	Gene Ratio	BgRatio	pvalue	p.adjust	qvalue	geneID	Count
GO:0007212	dopamine receptor signaling pathway	4/43	37/16672	2.38E-06	0.002402473	0.001887291	PALM/GNA15/GNA11/KLF16	4
GO:0003009	skeletal muscle contraction	3/43	38/16672	0.000126592	0.041598317	0.032678037	EEF2/TCAP/MYH8	3
GO:0050879	multicellular organismal movement	3/43	47/16672	0.000239423	0.041598317	0.032678037	EEF2/TCAP/MYH8	3
GO:0050881	musculoskeletal movement	3/43	47/16672	0.000239423	0.041598317	0.032678037	EEF2/TCAP/MYH8	3
GO:006	trabec	3/43	48/166	0.0002	0.0415	0.0326	MED1/S	3

1383	ula morphogenesis		72	54926	98317	78037	BNO2/UBE4B	
GO:0006352	DNA-templated transcription, initiation	5/43	228/16672	0.000288531	0.041598317	0.032678037	MED1/MED16/POLR2E/MED24/POLRMT	5
GO:0060158	phospholipase C-activating dopamine receptor signaling pathway	2/43	10/16672	0.000288591	0.041598317	0.032678037	GNA15/GNA11	2
GO:0035747	natural killer cell chemotaxis	2/43	11/16672	0.000352145	0.044414239	0.034890117	CCL7/CCL4	2
GO:0010839	negative regulation of keratinocyte proliferation	2/43	13/16672	0.000497771	0.048427142	0.0380425	MED1/KDF1	2
GO:0030521	androgen receptor	3/43	62/16672	0.0005436	0.048427142	0.0380425	MED1/MED16/MED24	3

	signaling pathway							
GO:0055008	cardiac muscle tissue morphogenesis	3/43	62/16672	0.0005436	0.048427142	0.0380425	MED1/TCAP/UBE4B	3
GO:0009314	response to radiation	6/43	421/16672	0.000691344	0.048427142	0.0380425	PPP1R1B/CCL7/GNA11/MYC/ECT2/UBE4B	6
GO:0050678	regulation of epithelial cell proliferation	5/43	281/16672	0.000748148	0.048427142	0.0380425	MED1/PDCD10/MYDGF/MYC/KDF1	5
GO:0003222	ventricular trabecula myocardium morphogenesis	2/43	16/16672	0.000762048	0.048427142	0.0380425	MED1/UBE4B	2
GO:0060415	muscle tissue morphogenesis	3/43	72/16672	0.000841995	0.048427142	0.0380425	MED1/TCAP/UBE4B	3
GO:0007213	G-protein coupled acetyl	2/43	18/16672	0.000968435	0.048427142	0.0380425	GNA15/GNA11	2

	choline receptor signaling pathway							
GO:0095500	acetylcholine receptor signaling pathway	2/43	18/16672	0.000968435	0.048427142	0.0380425	GNA15/GNA11	2
GO:1903831	signal transduction involved in cellular response to ammonium ion	2/43	18/16672	0.000968435	0.048427142	0.0380425	GNA15/GNA11	2
GO:1905144	response to acetylcholine	2/43	18/16672	0.000968435	0.048427142	0.0380425	GNA15/GNA11	2
GO:1905145	cellular response to acetylcholine	2/43	18/16672	0.000968435	0.048427142	0.0380425	GNA15/GNA11	2
GO:0006367	transcription initia	4/43	173/16672	0.001007899	0.048427142	0.0380425	MED1/MED16/POLR2E/MED24	4

	tion from RNA polymerase II promoter							
GO:0048644	muscle organ morphogenesis	3/43	78/16672	0.001062553	0.048732561	0.038282425	MED1/TCAP/UBE4B	3
GO:0045606	positive regulation of epidermal cell differentiation	2/43	20/16672	0.001198702	0.049139845	0.038602372	MED1/KDF1	2
GO:1901623	regulation of lymphocyte chemotaxis	2/43	20/16672	0.001198702	0.049139845	0.038602372	CCL7/CCL4	2
GO:0043410	positive regulation of MAPK cascade	6/43	471/16672	0.001236736	0.049139845	0.038602372	CCL7/PDCD10/PIK3R5/GADD45B/MYDGF/CCL4	6
GO:0002573	myeloid leukocyte differentiation	4/43	184/16672	0.0012 6624	0.049139845	0.038602372	MED1/FSTL3/MYC/SBNO2	4
GO:0071242	cellular	2/43	21/16672	0.001322715	0.049430352	0.038830583	GNA15/GNA11	2

	response to ammonium ion							
GO:0060359	response to ammonium ion	2/43	22/16672	0.001452608	0.052345774	0.041120826	GNA15/GNA11	2
GO:0014706	striated muscle tissue development	5/43	329/16672	0.001510638	0.052559774	0.041288936	MED1/EEF2/TCAP/HMG20B/UBE4B	5
GO:0050878	regulation of body fluid levels	6/43	495/16672	0.001594166	0.053617128	0.042119553	MED1/PIK3R5/GNA15/GNA11/HMG20B/KDF1	6
GO:0050673	epithelial cell proliferation	5/43	336/16672	0.001657253	0.05394091	0.042373904	MED1/PDCD10/MYDGF/MYC/KDF1	5
GO:0060537	muscle tissue development	5/43	343/16672	0.001814111	0.055710777	0.043764243	MED1/EEF2/TCAP/HMG20B/UBE4B	5
GO:0007517	muscle organ development	5/43	344/16672	0.00183738	0.055710777	0.043764243	MED1/EEF2/TCAP/HMG20B/UBE4B	5
GO:2000403	positive regulation of lymphocyte migration	2/43	25/16672	0.001877271	0.055710777	0.043764243	CCL7/CCL4	2

GO:0007200	phospholipase C-activating G-protein coupled receptor signaling pathway	3/43	98/16672	0.002049623	0.059087706	0.046417029	KISS1R/GNA15/GNA11	3
GO:0010837	regulation of keratinocyte proliferation	2/43	29/16672	0.002524017	0.070742591	0.055572658	MED1/KDF1	2
GO:0030224	monocyte differentiation	2/43	31/16672	0.002881378	0.074546413	0.058560794	MED1/MYC	2
GO:0060603	mammary gland duct morphogenesis	2/43	31/16672	0.002881378	0.074546413	0.058560794	MED1/SOSTDC1	2
GO:1903131	mononuclear cell differentiation	2/43	31/16672	0.002881378	0.074546413	0.058560794	MED1/MYC	2
GO:0045684	positive regulation of epider	2/43	32/16672	0.003068454	0.077401745	0.060803833	MED1/KDF1	2

	mis development							
GO:0050680	negative regulation of epithelial cell proliferation	3/43	115/16672	0.003227682	0.078343773	0.061543853	MED1/PDCD10/KDF1	3
GO:0061384	heart trabecula morphogenesis	2/43	33/16672	0.003261089	0.078343773	0.061543853	MED1/UBE4B	2
GO:0042542	response to hydrogen peroxide	3/43	119/16672	0.00355404	0.083395955	0.065512653	PDCD10/EEF2/ECT2	3
GO:0030518	intracellular steroid hormone receptor signaling pathway	3/43	123/16672	0.003899928	0.089432444	0.070254687	MED1/MED16/MED24	3
GO:2000401	regulation of lymphocyte migration	2/43	38/16672	0.004306651	0.093323089	0.073311028	CCL7/CCL4	2

GO:0071248	cellular response to metal ion	3/43	129/16672	0.004456151	0.093323089	0.073311028	MT1M/FSTL3/ECT2	3
GO:0030049	muscle filament sliding	2/43	39/16672	0.004532043	0.093323089	0.073311028	TCAP/MYH8	2
GO:0033275	actin-myosin filament sliding	2/43	39/16672	0.004532043	0.093323089	0.073311028	TCAP/MYH8	2
GO:0043616	keratinocyte proliferation	2/43	39/16672	0.004532043	0.093323089	0.073311028	MED1/KDF1	2
GO:0051302	regulation of cell division	3/43	132/16672	0.004751394	0.095883123	0.075322092	MYC/ECT2/KDF1	3

S7 Table. The pathways KEGG pathways enriched by DRGs.

Pathway ID	Description	GeneRatio	BgRatio	pvalue	p.adjust	qvalue	geneID	Count
hsa04062	Chemokine signaling pathway	6/19	182/7240	4.80E-06	0.000240129	0.000240129	6356/6360/6362/6368/6355/6352	6
hsa04060	Cytokine-cytokine receptor intera	6/19	270/7240	4.58E-05	0.001145446	0.001145446	6356/6360/6362/6368/6355/6352	6

	ction							
hsa05012	Parkinson's disease	2/19	142/7240	0.052536778	0.355317868	0.355317868	4711/6570	2
hsa05224	Breast cancer	2/19	144/7240	0.053865778	0.355317868	0.355317868	672/27006	2
hsa00514	Other types of O-glycan biosynthesis	1/19	22/7240	0.056250789	0.355317868	0.355317868	4242	1
hsa03010	Ribosome	2/19	154/7240	0.060690862	0.355317868	0.355317868	6209/6143	2
hsa04977	Vitamin digestion and absorption	1/19	24/7240	0.061213071	0.355317868	0.355317868	338	1
hsa04950	Maturity onset diabetes of the young	1/19	26/7240	0.066150627	0.355317868	0.355317868	6928	1
hsa04621	NOD-like receptor signaling pathway	2/19	168/7240	0.070723763	0.355317868	0.355317868	55669/6352	2
hsa04744	Phototransduction	1/19	28/7240	0.071063574	0.355317868	0.355317868	5957	1
hsa05310	Asthma	1/19	31/7240	0.078387105	0.356305023	0.356305023	6356	1
hsa05020	Prion diseas	1/19	35/7240	0.088066759	0.362050403	0.362050403	6352	1

	es							
hsa03440	Homologous recombination	1/19	41/7240	0.102405834	0.362050403	0.362050403	672	1
hsa04975	Fat digestion and absorption	1/19	41/7240	0.102405834	0.362050403	0.362050403	338	1
hsa04330	Notch signaling pathway	1/19	48/7240	0.118864779	0.362050403	0.362050403	4242	1
hsa05030	Cocaine addiction	1/19	49/7240	0.121192583	0.362050403	0.362050403	6570	1
hsa03460	Fanconi anemia pathway	1/19	55/7240	0.135037528	0.362050403	0.362050403	672	1
hsa04721	Synaptic vesicle cycle	1/19	63/7240	0.153176258	0.362050403	0.362050403	6570	1
hsa04623	Cytosolic DNA-sensing pathway	1/19	64/7240	0.155418093	0.362050403	0.362050403	6352	1
hsa04137	Mitophagy - animal	1/19	65/7240	0.157654305	0.362050403	0.362050403	55669	1
hsa05031	Amphetamine addiction	1/19	68/7240	0.164329334	0.362050403	0.362050403	6570	1
hsa05120	Epithelial	1/19	68/7240	0.164329334	0.362050403	0.362050403	6352	1

	cell signaling in Helicobacter pylori infection							
hsa05218	Melanoma	1/19	69/7240	0.166543185	0.362050403	0.362050403	27006	1
hsa01524	Platinum drug resistance	1/19	73/7240	0.175343153	0.365298235	0.365298235	672	1
hsa04911	Insulin secretion	1/19	85/7240	0.201217945	0.40103239	0.40103239	27094	1
hsa05323	Rheumatoid arthritis	1/19	90/7240	0.211770473	0.40103239	0.40103239	6352	1
hsa04657	IL-17 signaling pathway	1/19	93/7240	0.218038452	0.40103239	0.40103239	6356	1
hsa04151	PI3K-Akt signaling pathway	2/19	342/7240	0.225536145	0.40103239	0.40103239	672/27006	2
hsa05142	Chagas disease (American trypanosomiasis)	1/19	102/7240	0.236560296	0.40103239	0.40103239	6352	1
hsa04620	Toll-like recept	1/19	104/7240	0.240619434	0.40103239	0.40103239	6352	1

	or signaling pathway							
hsa04668	TNF signaling pathway	1/19	108/7240	0.248676451	0.401091051	0.401091051	6352	1
hsa04726	Serotonergic synapse	1/19	113/7240	0.258633864	0.404115412	0.404115412	6570	1
hsa04270	Vascular smooth muscle contraction	1/19	121/7240	0.274306115	0.415615326	0.415615326	27094	1
hsa04728	Dopaminergic synapse	1/19	130/7240	0.291562105	0.423213772	0.423213772	6570	1
hsa00190	Oxidative phosphorylation	1/19	133/7240	0.297227196	0.423213772	0.423213772	4711	1
hsa04120	Ubiquitin mediated proteolysis	1/19	137/7240	0.304713916	0.423213772	0.423213772	672	1
hsa04932	Non-alcoholic fatty liver disease (NAFLD)	1/19	149/7240	0.326723345	0.441518034	0.441518034	4711	1
hsa040	cGMP-P	1/19	163/72	0.3515	0.4497	0.4497	27094	1

22	KG signaling pathway		40	6672	28714	28714		
hsa04530	Tight junction	1/19	170/7240	0.363660278	0.449728714	0.449728714	4620	1
hsa05010	Alzheimer's disease	1/19	171/7240	0.365370385	0.449728714	0.449728714	4711	1
hsa05164	Influenza A	1/19	173/7240	0.368777545	0.449728714	0.449728714	6352	1
hsa05034	Alcoholism	1/19	180/7240	0.380566645	0.452162082	0.452162082	6570	1
hsa05168	Herpes simplex infection	1/19	185/7240	0.388859391	0.452162082	0.452162082	6352	1
hsa05016	Huntington's disease	1/19	193/7240	0.401909386	0.456715212	0.456715212	4711	1
hsa04015	Rap1 signaling pathway	1/19	210/7240	0.428769386	0.469405614	0.469405614	27006	1
hsa04810	Regulation of actin cytoskeleton	1/19	212/7240	0.431853165	0.469405614	0.469405614	27006	1
hsa04014	Ras signaling pathway	1/19	227/7240	0.454483883	0.483493493	0.483493493	27006	1
hsa04010	MAPK signaling pathwa	1/19	255/7240	0.494461866	0.515064444	0.515064444	27006	1

	y							
hsa05206	MicroRNAs in cancer	1/19	299/7240	0.551726105	0.562985821	0.562985821	672	1
hsa05200	Pathways in cancer	1/19	395/7240	0.656070145	0.656070145	0.656070145	27006	1

S8 Table. The pathways KEGG pathways enriched by DSGs.

Pathway ID	Description	Gene Ratio	BgRatio	pvalue	p.adjust	qvalue	geneID	Count
hsa04919	Thyroid hormone signaling pathway	4/23	116/7240	0.000437833	0.032837463	0.031339614	5469/10025/9862/4609	4
hsa04210	Apoptosis	3/23	138/7240	0.009077066	0.340389971	0.324863411	4616/8743/84823	3
hsa04623	Cytosolic DNA-sensing pathway	2/23	64/7240	0.017271045	0.431776126	0.412081074	5434/6351	2
hsa04064	NF-kappa B signaling pathway	2/23	95/7240	0.036047657	0.445087718	0.424785471	4616/6351	2
hsa05146	Amoebiasis	2/23	96/7240	0.036744397	0.445087718	0.424785471	2769/2767	2
hsa05142	Chagas disease (American trypanosomia	2/23	102/7240	0.041034096	0.445087718	0.424785471	2769/2767	2

	sis)							
hsa04725	Cholinergic synapse	2/23	112/7240	0.048583264	0.445087718	0.424785471	23533/2767	2
hsa04060	Cytokine-cytokine receptor interaction	3/23	270/7240	0.052369957	0.445087718	0.424785471	6354/6351/8743	3
hsa04110	Cell cycle	2/23	124/7240	0.058258204	0.445087718	0.424785471	4616/4609	2
hsa04068	FoxO signaling pathway	2/23	132/7240	0.065054322	0.445087718	0.424785471	4616/8743	2
hsa04120	Ubiquitin mediated proteolysis	2/23	137/7240	0.069433695	0.445087718	0.424785471	997/10277	2
hsa04310	Wnt signaling pathway	2/23	143/7240	0.074816192	0.445087718	0.424785471	4609/79718	2
hsa04921	Oxytocin signaling pathway	2/23	153/7240	0.084078467	0.445087718	0.424785471	23533/1938	2
hsa05216	Thyroid cancer	1/23	29/7240	0.088307851	0.445087718	0.424785471	4609	1
hsa04022	cGMP-PKG signaling pathwa	2/23	163/7240	0.093679605	0.445087718	0.424785471	23533/2767	2

	y							
hsa03020	RNA polymerase	1/23	32/7240	0.09700498	0.445087718	0.424785471	5434	1
hsa05202	Transcriptional misregulation in cancer	2/23	180/7240	0.110700191	0.445087718	0.424785471	4609/84444	2
hsa04020	Calcium signaling pathway	2/23	182/7240	0.112755555	0.445087718	0.424785471	2769/2767	2
hsa04062	Chemokine signaling pathway	2/23	182/7240	0.112755555	0.445087718	0.424785471	6354/6351	2
hsa05219	Bladder cancer	1/23	41/7240	0.122622775	0.459835406	0.438860458	4609	1
hsa05169	Epstein-Barr virus infection	2/23	203/7240	0.134928902	0.459926404	0.438947305	5434/4609	2
hsa05030	Cocaine addiction	1/23	49/7240	0.144809258	0.459926404	0.438947305	84152	1
hsa05213	Endometrial cancer	1/23	50/7240	0.147544536	0.459926404	0.438947305	4609	1
hsa04978	Mineral absorption	1/23	51/7240	0.150271445	0.459926404	0.438947305	4499	1
hsa05221	Acute myeloi	1/23	55/7240	0.161095875	0.459926404	0.438947305	4609	1

	d leukemia							
hsa00310	Lysine degradation	1/23	59/7240	0.171788338	0.459926404	0.438947305	84444	1
hsa04730	Long-term depression	1/23	60/7240	0.174441014	0.459926404	0.438947305	2767	1
hsa05210	Colorectal cancer	1/23	60/7240	0.174441014	0.459926404	0.438947305	4609	1
hsa05230	Central carbon metabolism in cancer	1/23	65/7240	0.18758295	0.459926404	0.438947305	4609	1
hsa04010	MAPK signaling pathway	2/23	255/7240	0.193419416	0.459926404	0.438947305	4616/4609	2
hsa05031	Amphetamine addiction	1/23	68/7240	0.195371806	0.459926404	0.438947305	84152	1
hsa04115	p53 signaling pathway	1/23	69/7240	0.19795218	0.459926404	0.438947305	4616	1
hsa05220	Chronic myeloid leukemia	1/23	71/7240	0.203089204	0.459926404	0.438947305	4609	1
hsa04925	Aldosterone synthesis and secret	1/23	82/7240	0.23078504	0.459926404	0.438947305	2767	1

	ion							
hsa04350	TGF-beta signaling pathway	1/23	84/7240	0.235720707	0.459926404	0.438947305	4609	1
hsa05222	Small cell lung cancer	1/23	84/7240	0.235720707	0.459926404	0.438947305	4609	1
hsa04911	Insulin secretion	1/23	85/7240	0.238177166	0.459926404	0.438947305	2767	1
hsa04012	ErbB signaling pathway	1/23	86/7240	0.240626073	0.459926404	0.438947305	4609	1
hsa05132	Salmonella infection	1/23	86/7240	0.240626073	0.459926404	0.438947305	6351	1
hsa04540	Gap junction	1/23	88/7240	0.245501315	0.459926404	0.438947305	2767	1
hsa04912	GnRH signaling pathway	1/23	92/7240	0.255162134	0.459926404	0.438947305	2767	1
hsa04657	IL-17 signaling pathway	1/23	93/7240	0.257558786	0.459926404	0.438947305	6354	1
hsa01522	Endocrine resistance	1/23	96/7240	0.264704566	0.460504114	0.439498663	5469	1
hsa04620	Toll-like recept	1/23	104/7240	0.283439793	0.460504114	0.439498663	6351	1

	or signaling pathway							
hsa00240	Pyrimidine metabolism	1/23	105/7240	0.285749334	0.460504114	0.439498663	5434	1
hsa04151	PI3K-Akt signaling pathway	2/23	342/7240	0.296744965	0.460504114	0.439498663	23533/4609	2
hsa05145	Toxoplasmosis	1/23	113/7240	0.303971052	0.460504114	0.439498663	23533	1
hsa04152	AMPK signaling pathway	1/23	121/7240	0.32174762	0.460504114	0.439498663	1938	1
hsa04270	Vascular smooth muscle contraction	1/23	121/7240	0.32174762	0.460504114	0.439498663	2767	1
hsa04611	Platelet activation	1/23	123/7240	0.32612343	0.460504114	0.439498663	23533	1
hsa04728	Dopaminergic synapse	1/23	130/7240	0.341227097	0.460504114	0.439498663	84152	1
hsa04650	Natural killer cell mediated cytoto	1/23	133/7240	0.347600486	0.460504114	0.439498663	8743	1

	xicity							
hsa03040	Spliceosome	1/23	134/7240	0.349711812	0.460504114	0.439498663	8175	1
hsa05162	Measles	1/23	134/7240	0.349711812	0.460504114	0.439498663	8743	1
hsa04371	Apelin signaling pathway	1/23	138/7240	0.35809194	0.460504114	0.439498663	23533	1
hsa05200	Pathways in cancer	2/23	395/7240	0.359727345	0.460504114	0.439498663	2767/4609	2
hsa04550	Signaling pathways regulating pluripotency of stem cells	1/23	139/7240	0.360170774	0.460504114	0.439498663	4609	1
hsa04261	Adrenergic signaling in cardiomyocytes	1/23	144/7240	0.370468721	0.460504114	0.439498663	23533	1
hsa05161	Hepatitis B	1/23	144/7240	0.370468721	0.460504114	0.439498663	4609	1
hsa05224	Breast cancer	1/23	144/7240	0.370468721	0.460504114	0.439498663	4609	1
hsa04072	Phospholipase D signaling pathway	1/23	146/7240	0.374543346	0.460504114	0.439498663	23533	1
hsa04390	Hippo signaling	1/23	154/7240	0.390591002	0.4696916	0.448267071	4609	1

	pathway							
hsa04630	Jak-STAT signaling pathway	1/23	156/7240	0.394540944	0.4696916	0.448267071	4609	1
hsa04141	Protein processing in endoplasmic reticulum	1/23	166/7240	0.413926109	0.482310396	0.460310273	10277	1
hsa04530	Tight junction	1/23	170/7240	0.421512726	0.482310396	0.460310273	4626	1
hsa05164	Influenza A	1/23	173/7240	0.427140947	0.482310396	0.460310273	8743	1
hsa00230	Purine metabolism	1/23	175/7240	0.430863954	0.482310396	0.460310273	5434	1
hsa05034	Alcoholism	1/23	180/7240	0.44007049	0.485371864	0.463232095	84152	1
hsa05168	Herpes simplex infection	1/23	185/7240	0.449134473	0.488189645	0.465921345	997	1
hsa05016	Huntington's disease	1/23	193/7240	0.463345587	0.4964417	0.473796991	5434	1
hsa04024	cAMP signaling pathway	1/23	198/7240	0.472048725	0.498643019	0.475897899	84152	1
hsa05205	Proteoglycans in cancer	1/23	203/7240	0.480616762	0.50064246	0.477806137	4609	1

hsa05166	HTLV-I infection	1/23	256/7240	0.563632079	0.579074054	0.55266015	4609	1
hsa04080	Neuroactive ligand-receptor interaction	1/23	278/7240	0.594222349	0.602252381	0.57478122	84634	1
hsa05206	MicroRNAs in cancer	1/23	299/7240	0.621500896	0.621500896	0.593151732	4609	1
